# Improving dynamic predictions with ensembles of observable models

**DOI:** 10.1093/bioinformatics/btac755

**Published:** 2022-11-23

**Authors:** Gemma Massonis, Alejandro F Villaverde, Julio R Banga

**Affiliations:** Computational Biology Lab, MBG-CSIC (Spanish National Research Council), Pontevedra, Galicia 36143, Spain; CITMAga, Santiago de Compostela, Galicia 15782, Spain; Department of Systems and Control Engineering, Universidade de Vigo, Vigo, Galicia 36310, Spain; Computational Biology Lab, MBG-CSIC (Spanish National Research Council), Pontevedra, Galicia 36143, Spain

## Abstract

**Motivation:**

Dynamic mechanistic modelling in systems biology has been hampered by the complexity and variability associated with the underlying interactions, and by uncertain and sparse experimental measurements. Ensemble modelling, a concept initially developed in statistical mechanics, has been introduced in biological applications with the aim of mitigating those issues. Ensemble modelling uses a collection of different models compatible with the observed data to describe the phenomena of interest. However, since systems biology models often suffer from a lack of identifiability and observability, ensembles of models are particularly unreliable when predicting non-observable states.

**Results:**

We present a strategy to assess and improve the reliability of a class of model ensembles. In particular, we consider kinetic models described using ordinary differential equations with a fixed structure. Our approach builds an ensemble with a selection of the parameter vectors found when performing parameter estimation with a global optimization metaheuristic. This technique enforces diversity during the sampling of parameter space and it can quantify the uncertainty in the predictions of state trajectories. We couple this strategy with structural identifiability and observability analysis, and when these tests detect possible prediction issues we obtain model reparameterizations that surmount them. The end result is an ensemble of models with the ability to predict the internal dynamics of a biological process. We demonstrate our approach with models of glucose regulation, cell division, circadian oscillations and the JAK-STAT signalling pathway.

**Availability and implementation:**

The code that implements the methodology and reproduces the results is available at https://doi.org/10.5281/zenodo.6782638.

**Supplementary information:**

Supplementary data are available at *Bioinformatics* online.

## 1 Introduction

Modelling and analysis of cellular networks under uncertainty remain a fundamental challenge in systems biology, biotechnology and bioengineering (Kaltenbach *et al.*, 2009; Mišković and Hatzimanikatis, 2011). Ensemble modelling, a concept initially developed in statistical mechanics (Brown and Sethna, 2003) that uses a collection of different models compatible with the observed data to describe the phenomena of interest, is a suitable strategy to handle a model’s parametric and structural uncertainty (Kirk *et al.*, 2015; Kremling *et al.*, 2018; Kuepfer *et al.*, 2007).

During the last two decades, the use of model ensembles has started to play an increasingly important role in the study of biological systems (Swigon, 2012), with applications in cell signalling (Brown and Sethna, 2003; Kuepfer *et al.*, 2007), metabolic networks (Hameri *et al.*, 2019; Jia *et al.*, 2012; Saa and Nielsen, 2017; Tran *et al.*, 2008) and gene expression and regulation (Samee *et al.*, 2015; Ud-Dean and Gunawan, 2014). However, building model ensembles is typically very computationally costly. Further, recent research has revealed that ensemble modelling can exhibit a number of important pitfalls (Stumpf, 2020), so ensembles must be carefully constructed in order to avoid them.

Here, we present a strategy to assess and improve the reliability of a class of model ensembles. In particular, we consider kinetic models described using ordinary differential equations (ODEs) with a fixed structure. In previous work (Villaverde *et al.*, 2015), we developed a consensus-based technique, where the ensemble is built using the sampling from optimization runs of parameter estimation by means of a global optimization metaheuristic that enforces diversity during the sampling of parameter space. This method was successfully used to perform uncertainty quantification of state predictions of large kinetic models (Villaverde *et al.*, 2022).

**Fig. 1.**
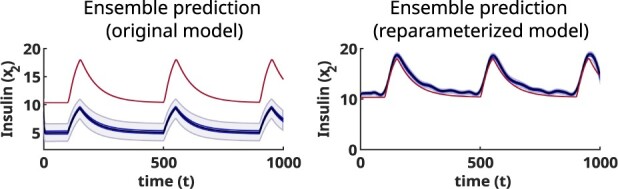
Illustration of the core idea behind our method. The plots show ensemble predictions of the time course of insulin concentration, produced by the *β*IG model of glucose regulation (for details, see Section 3, Table 1 and the Supplementary Information). The true trajectory is shown as a red line, the ensemble prediction as a black line, and the darker and lighter blue shaded areas represent the 40% and 80% confidence percentiles, respectively. The left-hand plot shows the ensemble prediction of the original model, in which for the state variable *x*_1_, corresponding to insulin concentration, is unobservable. This model has three states (of which only one, glucose, is measured; the two unmeasured states are unobservable) and five parameters (two of which are unidentifiable). Due to the lack of structural identifiability and observability, the ensemble prediction does not reproduce the true trajectory. The right-hand plot shows the ensemble prediction of the reparameterized model, which is fully observable. As a result of insulin becoming observable, the new simulations of its time course are much closer to the true trajectory, and the confidence intervals are more accurate representations of the prediction uncertainty. The NRMSE for the original model (unobservable) is 0.8516, while the NRMSE for the reparameterized model (observable) is 0.1455, a reduction of 82.91%

Our new contribution is based on the observation that most models in systems biology suffer from a lack of distinguishability, identifiability and observability (Kreutz *et al.*, 2012; Szederkényi *et al.*, 2011; Wieland *et al.*, 2021). As a consequence, we expect ensembles of models to be particularly unreliable when predicting non-observable states. In order to surmount these difficulties, we present a new methodology that starts by analysing structural identifiability and observability. When these analyses reveal deficiencies in the model structure that could lead to prediction issues, our method searches for model reformulations that surmount those difficulties. Once a fully identifiable and observable model structure has been obtained, we perform parameter estimation and use the results to build an ensemble of models following a systematic procedure described in this article. The resulting ensemble allows making predictions about the time course of internal (i.e. unmeasured) state variables, as well as quantifying their uncertainty. Figure 1 illustrates the core idea by means of the glucose regulation model, where the lack of identifiability and observability is surmounted by merging some of the non-identifiable parameters into new variables, yielding a fully observable model.

In summary, in this article, we address three key issues. The first one is the analysis of the role of structural identifiability and observability in the context of ensemble modelling, a previously overlooked topic. Second, the comparison of the uncertainty in the predictions made by ensembles of observable models versus unobservable models. Third, the development of a step-by-step procedure following a frequentist approach, where each step is clearly described and mathematically formulated.

## 2 Materials and methods

**Fig. 2.**
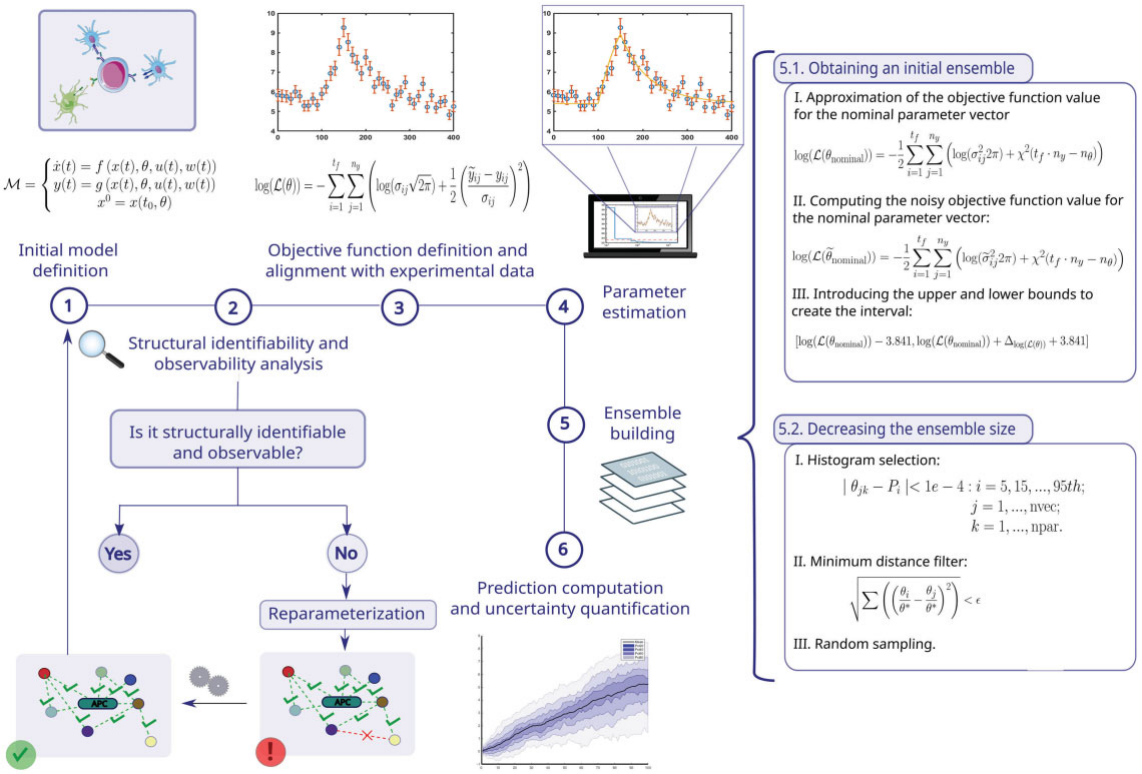
Outline of the proposed ensemble modelling methodology

The proposed methodology consists of the six main steps shown in Figure 2. We describe them in the following subsections. Furthermore, we provide their pseudo-code as a Supplementary Information file.

### 2.1 Model definition

We consider dynamic models described by systems of deterministic ODEs of the form:

$$M=\{\begin{array}{c} \dot{x}(t)=f\left(x(t),\theta ,u(t),w(t)\right)\;(1)\\ y(t)=g\left(x(t),\theta ,u(t),w(t)\right)\;\;\;\;\;(2)\\ x^{0}=x(t_{0},\theta )\;\;\;\;\;\;\;(3) \end{array}$$

where *f* and *g* are rational functions of the states, $x(t)\in R^{n_{x}},$ known and unknown inputs, $u(t)\in R^{n_{u}}$ and $w(t)\in R^{n_{w}}$, respectively, and unknown constant parameters, $\theta \in R^{n_{\theta }}$. The output, $y(t)\in R^{n_{y}}$, represents the measurements as functions of model variables.

### 2.2 Structural identifiability and observability: analysis and reparameterization

Once a dynamic model is available, we analyse its *structural* identifiability and observability. This analysis reveals if it is possible to estimate the true values of the parameters *θ* (identifiability), state variables *x*(*t*) (observability) and unknown inputs *w*(*t*) (input observability) from output measurements, *y*(*t*) (Distefano, 2015).

If this analysis yields a positive result, the model is said to have the FISPO property, which stands for Full Input-State-Parameter Observability (or Observable, when the acronym is used as an adjective). This property, along with a method for its analysis and an implementation in the Matlab toolbox STRIKE-GOLDD (https://github.com/afvillaverde/strike-goldd), was introduced by Villaverde *et al.* (2019b). Other tools can be used to analyse structural identifiability and observability, based on differential geometry or differential algebra.

If the analysis yields a negative result, the model is unidentifiable and/or unobservable. This means that its outputs are invariant under certain modifications of some of its parameters and/or states. This can be explained mathematically as the existence of Lie symmetries among model variables (Merkt *et al.*, 2015). If it is possible to find and remove such symmetries by reparameterization, a FISPO model can be achieved. To this end, we use AutoRepar (Massonis *et al.*, 2021), a method included in the aforementioned Matlab toolbox STRIKE-GOLDD, which computes model reformulations that seek to preserve the mechanistic character of selected model variables.

It should be noted that, even if a model is FISPO, it may still produce bad parameter estimates if its *practical* identifiability is poor. Practical identifiability analysis takes into account the quantity and quality of the available data (Wieland *et al.*, 2021). It can be performed with a number of methods, including the Fisher Information Matrix, profile likelihood and sampling-based procedures. Practical unidentifiability may be surmounted by using additional experimental data, ideally obtained from optimal experimental designs.

Although it is not the focus of the present article, the methodology could be extended so as to address practical identifiability issues. To this end, an additional procedure might be added between Steps 4 and 5. It would consist of an analysis of practical identifiability, possibly followed by an improved experiment design to obtain more time-point measurements and/or data of better quality, and the repetition of the procedure since the parameter estimation step.

### 2.3 Objective function definition

Parameter estimation, also known as model calibration, consists of finding the set of parameters that provide the optimal fit between model predictions and experimental data. It is performed by optimizing an objective function that quantifies the distance between data and model output (Villaverde *et al.*, 2021).

The likelihood of observing the data *D* given parameters *θ*, assuming independent, normally distributed additive measurements noise with standard deviation $\sigma_{i,j}$, is

$$P(D|\theta )=\prod_{i=1}^{t_{f}}\prod_{j=1}^{n_{y}}\frac{1}{\sqrt{2\pi }\sigma_{ij}}\;\text{exp}\;\left(-\frac{1}{2}{\left(\frac{{\tilde{y}}_{ij}-y_{ij}}{\sigma_{ij}}\right)}^{2}\right),$$

which corresponds to a Gaussian probability function. Maximizing this expression is equivalent to minimizing the negative log likelihood:

(4) $$-\text{log}(L(\theta ))=\sum_{i=1}^{t_{f}}\sum_{j=1}^{n_{y}}\left(\text{log}(\sigma_{ij}\sqrt{2\pi })+\frac{1}{2}{\left(\frac{{\tilde{y}}_{ij}-y_{ij}}{\sigma_{ij}}\right)}^{2}\right)$$

where $\tilde{y}$ is the measured data, and *σ_ij_* is the standard deviation of output *j* at the measurement time point *i*.

We use simulated data, so that the true solution is known and it is possible to assess the performance of our method. To this end, we generate synthetic data by adding normally distributed noise to the model outputs:

(5) $$\tilde{y}=y+\sigma =y+(\sigma_{\text{abs}}+\sigma_{\text{rel}}\cdot y)\cdot X,$$

where X is a normally distributed random number.If the noise distribution is unknown, the terms $\sigma_{\text{abs}}$ and $\sigma_{\text{rel}}$ must be included in the set of parameters to be optimized. Otherwise, they can be left out of the optimization.

### 2.4 Parameter estimation

Next, we perform parameter estimation by minimizing the objective function with numerical optimization methods. The goal of this step is not only to find the optimal solution of the parameter estimation problem (i.e. the optimal parameter vector and, in some cases, the $\sigma_{\text{rel}}$ and $\sigma_{\text{abs}}$) but also to obtain additional parameter vectors that will be used to build the ensemble. To this end, it is necessary to store the parameter vectors explored during the optimization procedure along with their objective function value.

Since, we wish to obtain an ensemble whose diversity represents the feasible ranges of parameter values, the optimization method must explore different regions of the parameter space. Purely local strategies are ill suited for this task, and global or hybrid optimization strategies should be used instead. In this work, we use an hybrid optimization approach implemented in the MEIGO Matlab toolbox (Egea *et al.*, 2014). It is a metaheuristic called enhanced scatter search (eSS), which is a population-based evolutionary optimization method that obtains new parameter vectors by pseudo-randomly exploring the parameter space, launching local searches from promising starting points, and recombining its members. As local search methods, we used the non-linear least-squares algorithm NL2SOL and the direct search method dynamic hill climbing. This hybrid approach has been shown to explore parameter spaces with multiple local minima in a computationally efficient way (Villaverde *et al.*, 2019a). In particular, the combination of diversification (global search) with intensification (local search) ensures a balanced sampling of the parameter space with results comparable to other sampling techniques such as MCMC (Villaverde *et al.*, 2022), and it avoids the biased sampling found in multi-start gradient-based methods (Fröhlich *et al.*, 2014). The sampling of (4) obtained during the optimization is stored for its post-processing in the next step.

### 2.5 Ensemble building

From the sampling of parameter vectors obtained during the optimization, we want to select a representative subset of those producing outputs in close agreement with the observed data. To this end, we select those that are inside a predefined admissible range of objective function values.

#### 2.5.1 Obtaining an initial ensemble

The goal of this step is to select parameter vectors with an objective function value similar to that of the true solution. Much higher values would indicate underfitting, and lower values overfitting. Since in practice the true value is unknown, we need to compute an approximation. Assuming that the errors are normally (Gaussian) distributed, the sum of squared residuals follows a $\chi^{2}$ distribution of $t_{f}\cdot n_{y}-n_{\theta }$ degrees of freedom, corresponding to the second term of the objective function (4) (Geier *et al.*, 2012). To approximate the first term of (4), we use the estimated values of the absolute and relative standard deviations, $\sigma_{abs^{*}}$ and $\sigma_{rel^{*}}$, obtaining

(6) $$\text{log}(L(\theta_{\text{nominal}}))=-\frac{1}{2}\sum_{i=1}^{t_{f}}\sum_{j=1}^{n_{y}}\left(\text{log}(\sigma_{ij}^{2}2\pi )+\chi^{2}(t_{f}\cdot n_{y}-n_{\theta })\right),$$

where $\sigma_{ij}=\sigma_{rel^{*}}\cdot y_{ij}+\sigma_{abs^{*}}$. Note that it is not necessary to know $\theta_{\text{nominal}}$ in order to calculate (6); instead, with this formula, we obtain an approximation of the log-likelihood value of the nominal parameter vector.

Next, we define an interval of values around this approximation. We set as lower bound the confidence region of $\text{log}(L(\theta_{\text{nominal}}))-\Delta_{\alpha }$, where $\Delta_{\alpha }$ is Pr$(\chi_{1}^{2})<0.05$ (i.e. 3.841). The term $\Delta_{\alpha }$ is the result of applying the *Likelihood Ratio Test* when all the parameters are fixed except *σ* (Vanlier *et al.*, 2012; Villaverde *et al.*, 2022). We found empirically that setting the upper bound at the same distance from $\text{log}(L(\theta_{\text{nominal}}))$ as the lower bound would lead to narrow confidence intervals, which would not always include the experimental data. Hence, we calculate the upper bound using a variant of equation (6), in which we modify the standard deviation ${\tilde{\sigma }}_{ij}$:

$$\text{log}(L({\tilde{\theta }}_{\text{nominal}}))=-\frac{1}{2}\sum_{i=1}^{t_{f}}\sum_{j=1}^{n_{y}}\left(\text{log}({\tilde{\sigma }}_{ij}^{2}2\pi )+\chi^{2}(t_{f}\cdot n_{y}-n_{\theta })\right),$$

where

$${\tilde{\sigma }}_{ij}=\sigma_{rel^{*}}\cdot y_{ij}\cdot \text{max}(\sigma_{\cdot ,j})+\sigma_{abs^{*}}\;.$$

Using the notation $\Delta_{\;\text{log}(L(\theta ))}=\text{log}(L({\tilde{\theta }}_{\text{nominal}}))-\text{log}(L(\theta_{\text{nominal}}))$, we obtain the following interval:

(7) $$\left[\text{log}(L(\theta_{\text{nominal}}))-3.841,\;\text{log}(L(\theta_{\text{nominal}}))+\Delta_{\;\text{log}(L(\theta ))}+3.841\right]$$

As a result of this step, every parameter vector that produces an objective function value in the interval (7) is included in the ensemble. This interval is an approximation based on a statistical criterion, namely the likelihood ratio test, as used by e.g. Vanlier *et al.* (2012) and Villaverde *et al.* (2022).

#### 2.5.2 Decreasing the ensemble size

There are typically a very large number of parameter vectors within the range defined by (7). To avoid excessive redundancy while preserving the desired diversity, we select a representative subset of them as follows.

First we compute, for each parameter, all percentiles from 0 to 100 with a step of 5 (giving a total of 21 groups). In order to reduce the total number of vectors while preserving the underlying distribution, we eliminate some of these groups based on its position inside the percentiles. Specifically, we keep only those that are in an even position. In this way, we discard the two extremes, leaving out the outliers while keeping values far from the mean, median and mode, which contain information not provided by measures of central tendency.

Second, to avoid almost identical vectors, we introduce a minimal distance criterion. To this end, we use the relative Euclidean distance re-scaled by the *best fit* ($\theta^{*}$) between a candidate vector (*θ_i_*) and all the others (*θ_j_*). We exclude a parameter vector from the ensemble if this distance is less than a cut-off value. That is, a vector *θ_i_* is removed from the ensemble if:

(8) $$\sqrt{\sum \left({\left(\frac{\theta_{i}}{\theta^{*}}-\frac{\theta_{j}}{\theta^{*}}\right)}^{2}\right)}<\epsilon$$

**Fig. 3.**
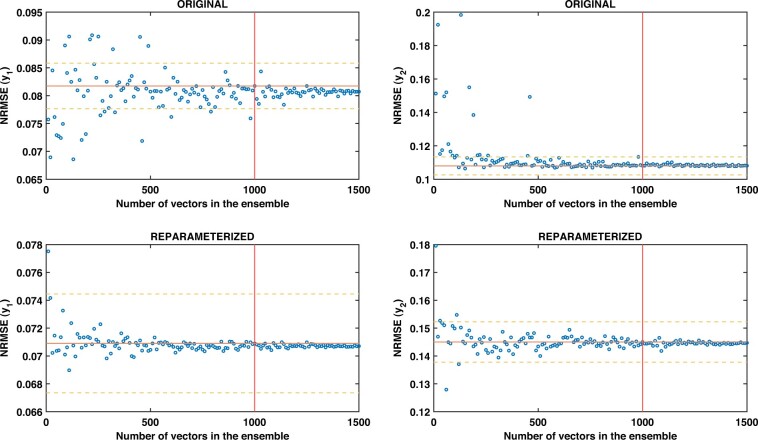
JAK-STAT: NRMSE (y) depending on the number of vectors considered for the ensemble. The orange line is the NRMSE of the ensemble considering 1000 vectors, while the yellow lines represent a variation of ±5% of it

Third, we perform a final reduction by random sampling. We have found empirically that ensembles of 1000 vectors are representative enough. Larger ensemble sizes do not increase the predictive power of the ensemble, as depicted in Figure 3 and discussed below.

**Fig. 4.**
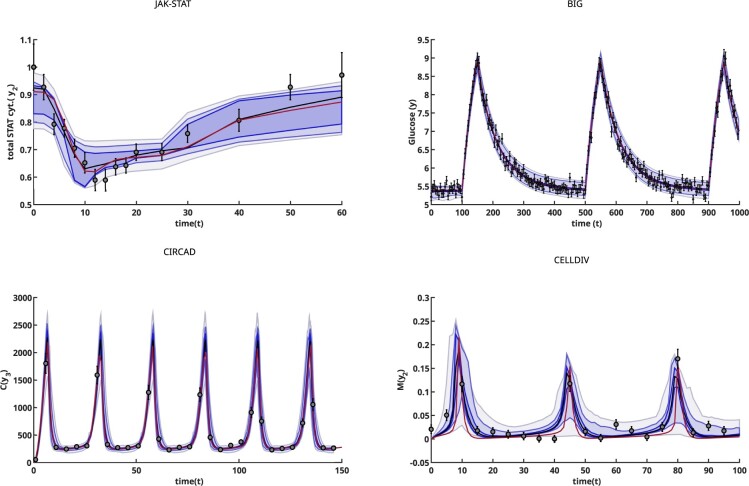
Illustration of the ensemble predictions for one output of each case study. The true trajectory is shown as a red line, the ensemble prediction as a black line, and the darker and lighter blue shaded areas represent the 2.2%, 50% and 97.5% confidence percentiles, respectively. Although the prediction accuracy varies among case studies, in all cases it can be noticed that the uncertainty envelope covers the experimental data

### 2.6 Ensemble prediction computation and uncertainty quantification

The ensemble prediction is defined as the median of the simulated model outputs using all the parameter vectors in the ensemble. The median is defined as the centrally placed value if these are ordered, or, equivalently, as the 50th percentile. It is an appropriate metric for unknown or non-Gaussian distributions, since it is less susceptible to outliers (Gneiting, 2011; Rousseeuw, 1990). The main metric for evaluating the quality of the ensemble predictions will be the normalized root mean square error (NRMSE), defined by the following expression:

(9) $$\text{NRMSE}(y)=\frac{\text{RMSE}(y^{f})}{\text{max}(\tilde{y})-\text{min}(\tilde{y})},$$

where

(10) $$\text{RMSE}(y^{f})=\sqrt{\frac{\sum_{i=1}^{n_{y}}{\left(y^{f}-\tilde{y}\right)}^{2}}{n_{y}}}.$$

denoting *y^f^* as the forecast ensemble. It is also possible to compute the NRMSE of internal states when the nominal parameter vector is known substituting, in the above expression, *y* and $\tilde{y}$ for *x* and $\tilde{x}$, respectively. Another variant of the above expression can be used to compute the NRMSE(*θ*) for the parameters. The NRMSE has the additional advantage of allowing the comparison of errors when model outputs, or parameters, have different orders of magnitude.

## 3 Results

In the present section, we demonstrate the proposed methodology by applying it to the models defined in Table 1, in order to illustrate all the aspects of the method and show its applicability. Here, we show only selected results of each case study; the accompanying repository (doi: 10.5281/zenodo.6782638) includes the full results, along with Matlab scripts that apply every step in the methodology.

**Table 1.** Main features of the case studies

ID	*β*IG	CELLDIV	CIRCAD	JAKSTAT
References	Karin *et al.* (2016)	Tyson (1991)	Vilar *et al.* (2002)	Vanlier *et al.* (2012)
Description	Glucose regulation	Cell division	Circadian oscillations	Signalling pathway
Parameters	5	8	15	6
Dynamic states	3	6	9	4
Observed states	1	2	3	2
Data points	200	20	30	16
Data type	Simulated	Simulated	Simulated	Real
Noise level	$\sigma_{\text{abs}}=2\%,\;\sigma_{\text{rel}}=2\%$	$\sigma_{\text{abs}}=2\%\;\sigma_{\text{rel}}=10\%$	$\sigma_{\text{abs}}=2\%\;\sigma_{\text{rel}}=10\%$	Real

### 3.1 Case studies

The models chosen for this study are described in Table 1. Their equations, as well as additional information about them, can be found in the scripts provided in the accompanying repository.

### 3.2 Structural identifiability and observability analysis and reparameterization

The observability and identifiability analyses concluded that none of the models are observable or identifiable. For the CELLDIV model, it was not possible to find a fully identifiable and observable reparameterization. In contrast, for the other three models (*βIG*, CIRCAD and JAKSTAT) the reparameterizations were as follows:

- The JAKSTAT model has three unidentifiable parameters and one unobservable state (*x*_1_, corresponding to the STAT variable). An identifiable reparameterization was found by transforming the unobservable variable so that it is multiplied by one of the unidentifiable parameters, *p*_2_. As a result, the following transformation was applied to *x*_1_: $x_{1}^{*}=x_{1}\cdot p_{2}$.
- The CIRCAD model has six unidentifiable parameters and two unobservable states (*M_A_* and *M_R_*). An identifiable reparameterization was found by transforming the two unobservable state variables, multiplying each of them by a parameter that is thus removed from the model. As a result, the following transformation was applied to *x*_5_ and *x*_7_: $x_{5}^{*}=x_{5}/\alpha_{AP}$ and $x_{7}^{*}=x_{7}/\alpha_{AR}$.
- The *β*IG model has two unobservable states (*x*_2_ and *x*_3_, corresponding to the insulin and *β* cell mass) and two unidentifiable parameters. In this case, an identifiable and observable reparameterization could be found, which makes the insulin state variable observable without undergoing any transformation. Instead, the transformations involve the other state (*x*_3_) and the unidentifiable parameters, which are removed from the model. The following reparameterization has been applied to *x*_3_ and the input *u*: $x_{3}^{*}=x_{3}\cdot p$ and $u^{*}=u-x_{3}\cdot x_{1}(s_{i}-x_{3})$. As a result, the unobservable state *x*_2_ became observable without undergoing any transformation.

### 3.3 Objective function definition

For each model, we defined the objective function as in (4), using simulated data. The values of $\sigma_{\text{abs}}$ and $\sigma_{\text{rel}}$ can be found in Table 1. Further details can be found in the accompanying scripts.

### 3.4 Parameter estimation

We performed parameter estimation for the four original models and the three reparameterized ones, by minimizing the objective function defined in the previous subsection, using a single-shooting approach (i.e. the dynamic model was simulated for each evaluation of the objective function). The optimizations were carried out using the eSS metaheuristic, as described in Section 2.4. The dynamic model simulations were performed with the AMICI toolbox (Fröhlich *et al.*, 2021) for all the cases except for the *β*IG model, for which the AMIGO2 toolbox (Balsa-Canto *et al.*, 2016) was used due to the need for estimating an unknown input.

### 3.5 Ensemble building

Following the procedure described in Section 2.5, we obtained ensembles of 1000 parameter vectors for each model. It should be noted that, while the optimizations had a fairly large computational cost—ranging from minutes for CELLDIV to days for CIRCAD (in this latter case, due to the challenging nature of parameter estimation in oscillatory models)—the process of ensemble building and exploitation took only a few minutes.

### 3.6 Prediction computation and uncertainty quantification: measured versus unmeasured states

The predictions of the *measured* state variables—i.e. the fits achieved from the optimization—obtained with both the original and the reparameterized model were of similar quality in all cases. For all models and versions, the ensemble trajectories approximated the experimental data, and their envelopes (which represent the prediction uncertainty) matched the standard deviation of the data, as shown in Figure 4.

**Fig. 5.**
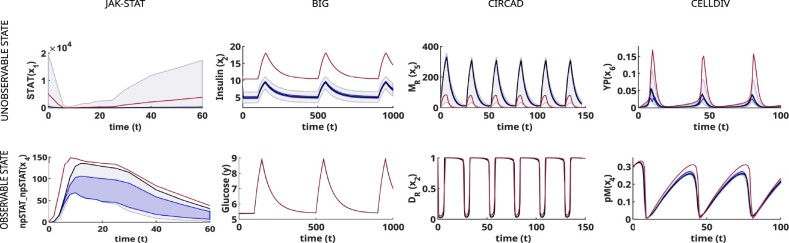
Illustration of the differences between ensemble predictions for an observable versus an unobservable state. For each case study (i.e. column), the figure shows time course simulations of one of the unobservable states (upper row) and one of the observable states (lower row). The true trajectory is shown as a red line, the ensemble prediction as a black line, and the darker and lighter blue shaded areas represent the 40% and 80% confidence percentiles, respectively. Although the prediction accuracy varies among case studies, in all cases it can be noticed that the predictions of unobservable states may be very far from reality, while predictions of observable states are better constrained and more precise

**Fig. 6.**
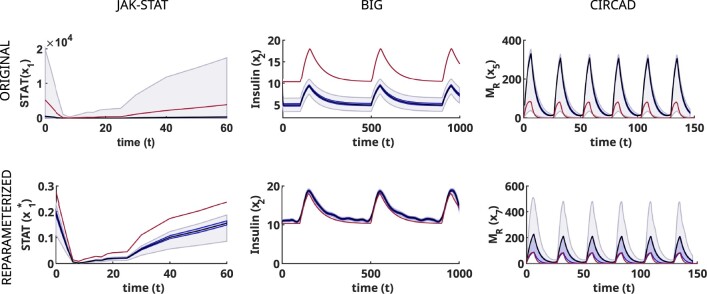
Using reparameterization, we transform an unobservable state variable (upper row) into an observable one (lower row), thus improving the quality of the corresponding ensemble predictions. Each column corresponds to a case study (for the fourth case study, CELLDIV, no observable reparameterization was found). The true trajectory is shown as a red line, the ensemble prediction as a black line, and the darker and lighter blue shaded areas represent the 40% and 80% confidence percentiles, respectively (A color version of this figure appears in the online version of this article)

In contrast, the results for the *internal—*i.e. not measured—states were clearly divided in two groups: observable and unobservable. The ensemble forecasts for the observable states were generally in good agreement with the true trajectories, i.e. those obtained when simulating the model with the nominal parameter vector. The uncertainty bounds were also close to these trajectories. Illustrative examples are shown in the lower row of Figure 5. For the unobservable states, on the other hand, the predicted trajectories usually showed large deviations from their true values (see the first rows of Figs 5 and 6), and in some cases, the prediction envelopes covered an overly large area (see the JAK-STAT example). For these cases, the ensemble predictions of the unobservable states were unreliable.

However, this limitation could be overcome by reparameterizing the models so that the unobservable states became observable (Fig. 6, second row). The ensemble forecasts produced by the reparameterized models were in better agreement with the true trajectories than the original predictions, and their NRMSEs decreased significantly. Furthermore, the reduction of the uncertainty bands in almost all cases is an indication of increased confidence in the predictions. These results illustrate the benefits of working with observable models. When ensemble modelling is applied to non-observable and non-identifiable models, the bounds of the predictions are only limited by the propagation to the state space of the parameter bounds set in the parameter estimation step. However, when the models are fully observable and identifiable, the ensemble forecast will be more narrowly constrained, as can be seen in Figures 5 and 6.

## 4 Discussion

In this work, we have studied the relevance of structural identifiability and observability for building and exploiting ensembles of dynamic models. As our results have shown, the lack of these properties can compromise predictive power. To address this issue, we have proposed an ensemble modelling framework that tests for the existence of such issues and takes the necessary actions to remedy them.

The procedure starts by analysing structural identifiability and observability; if the analysis of these properties reveals deficiencies in the model structure that prevent it from inferring key parameters or state variables, the method then searches for a suitable reparameterization. Once a fully identifiable and observable model structure is obtained, it is calibrated using a global optimization procedure that yields not only an optimal parameter vector but also an ensemble of other possible solutions. Our method exploits the information in these additional vectors to build an ensemble of models with different parameterizations. To this end, we have described how to select parameter vectors with appropriate objective function values, obtaining an ensemble of moderate size. The hybrid global optimization approach used here performs a balanced sampling of the parameter space; as a consequence, the median of the ensemble is a good approximation of the median of the model given parameter uncertainty. Furthermore, Villaverde *et al.* (2022) show how a parameter sampling similar to the one applied here yields good estimates of the uncertainty of the predictions.

The whole procedure can be performed systematically and is computationally efficient. To demonstrate its application, we have used four case studies based on models of different sizes, all of which have unobservable parameters and states. For each model, we created an ensemble that explains the available experimental data. However, when the ensembles are built from the initial models, their predictions of the unobservable internal states have high uncertainty. In contrast, if the ensembles are built from the reparameterized models, their predictions are better constrained. Importantly, in some cases, the reparameterization requires transforming all the unidentifiable parameters and all the unobservable state variables, while in other cases it is possible for certain variables of interest to become observable without being transformed. In the latter case, the mechanistic meaning of the untransformed variables is preserved, as was shown for the variable representing insulin concentration in the *β*IG model example.

In regard to the scalability of the approach, its main bottleneck is currently the reparameterization step. It is a task that involves symbolic computations, and its computational cost increases rapidly with model complexity. Massonis *et al.* (2021) applied it to a NF-*κ*B model of 30 variables; applying it to larger and/or more complex models can be challenging. However, it should be noted that the algorithmic improvements included in recent versions of the STRIKE-GOLDD toolbox have reduced its computational cost (Díaz-Seoane *et al.*, 2022). The other steps in our procedure have better scalability, and they can be applied to models with hundreds of variables, as shown in Villaverde *et al.* (2022).

## Supplementary Material

btac755_Supplementary_Data

## Data Availability

The data underlying this article are available in Zenodo, at https://dx.doi.org/10.5281/zenodo.6782638.
