# Supplementary material for: Improving dynamic predictions with ensembles of observable models
Source: Bioinformatics. 2022 Nov 23;39(1):btac755. doi: 10.1093/bioinformatics/btac755 (PMC9805594; doi:10.1093/bioinformatics/btac755)
Supplement: btac755_Supplementary_Data [file btac755_supplementary_data.pdf]

SUPPLEMENTARY INFORMATION OF THE PAPER
Improving dynamic predictions with ensembles of observable models

Gemma Massonis [1], Alejandro F. Villaverde [2,3] and Julio R. Banga [1]
[1]Computational Biology Lab, MBG-CSIC, Pontevedra, Galicia, Spain,
[2]CITMAga, Santiago de Compostela, Galicia, Spain,
[3]Universidade de Vigo, Dept. of Systems and Control Engineering, Vigo, Galicia, Spain
j.r.banga@csic.es, afvillaverde@uvigo.gal

June 30, 2022

The paper “Improving dynamic predictions with ensembles of observable models” presents a method for building ensembles of dynamic models. This document includes supplementary information to the main text of the paper, providing pseudo-code of the algorithms of each step in the methodology (in Section 1) and a brief description of the contents and location of the accompanying code (in Section 2).

1 Pseudocode of the algorithms

Algorithm 1 Initial model definition (Step 1)

Define the parameters $\longrightarrow \theta$
Define the states $\longrightarrow x(t)$
Define the inputs $\longrightarrow u(t), w(t)$
Define the equations $\longrightarrow \dot{x}(t) = f(x(t), \theta, u(t), w(t))$
Define the ICS $\longrightarrow x(t_0, \theta)$
Define the outputs $\longrightarrow y(t) = g(x(t), \theta, u(t), w(t))$
Define nº optimizations $\longrightarrow$ N_opt

Algorithm 2 Structural identifiability and observability analysis (Step 2)

Compile the model with *STRIKE-GOLDD*
if all the parameters are identifiable **then**
    Compile the model with AMICI
    Skip to step 3
else
    Use AutoRepar to obtain an identifiable reparameterization
    Compile the new model with AMICI
end if

Algorithm 3 Objective function definition and alignment with experimental data (Step 3)

Compute synthetic data $\longrightarrow \widetilde{y} = y + (\sigma_{\text{abs}} + \sigma_{\text{rel}} \cdot y) \cdot randn$
Compute the standard deviation $\sigma_y \longrightarrow \sigma_{\text{rel}}(1 + \widetilde{y})$
$t_f \longrightarrow$ total number of time points
$n_y \longrightarrow$ total number of outputs
Define the objective function $\longrightarrow \log(\mathcal{L}(\theta)) = \sum_{i=1}^{t_f} \sum_{j=1}^{n_y} \left(\log(\sigma_{ij}\sqrt{2\pi}) + \frac{1}{2} \left(\frac{\widetilde{y}-y}{\sigma_{ij}} \right)^2 \right)$

Algorithm 4 Parameter estimation (Step 4)

Definition of parameter bounds
Definition of solver and its options
for $i = 1, ..., \text{N\_opt}$ **do**
    Optimization of the objection function
    Save the best result $\longrightarrow$ Best fit
    Save all the attempts before reaching the best fit $\longrightarrow \text{par\_ array}_i$
end for

Algorithm 5 Obtaining an initial ensemble (Step 5.1)

Concatenate the four par_array $\longrightarrow$ *pvec*
Sort *pvec* in descending order of its objective function value
$n_\theta \longrightarrow$ total number of parameters
Obtain the σ^*_{rel} and σ^*_{abs} from the Best Fit
Compute the output of the model with the Best Fit parameters $\longrightarrow y^*$
Guess the objective function value for the nominal parameter vector $\longrightarrow$ nominal objf estim. $= -\frac{1}{2}\sum_{i=1}^{t_f}\sum_{j=1}^{n_y}\left(\log(\sigma_{ij}^2 2\pi) + \left(\chi^2(t_f \cdot n_y - n_\theta)\right)^2\right)$
Add noise to the $\sigma \longrightarrow \widetilde{\sigma}_{i,j} = \sigma^*_{\text{rel}} \cdot y^* \cdot \max(\sigma_y) + \sigma^*_{\text{abs}}$
Compute the noisy objective function $\longrightarrow$ noisy objf $= -\frac{1}{2}\sum_{i=1}^{t_f}\sum_{j=1}^{n_y}\left(\log((\sigma^*_{ij})^2 2\pi) + \left(\chi^2(t_f \cdot n_y - n_\theta)\right)^2\right)$
Define the upper bound $\longrightarrow UB =$ noisy objf $+ Pr(\chi^2_1) < 0.05$
Define the lower bound $\longrightarrow LB =$ nominal objf estim. $- Pr(\chi^2_1) < 0.05$
Select from *pvec*, those vectors that have an objf less than UB$\longrightarrow pvec1 = pvec < UB$
Select from *pvec1*, those vectors that have an objf higher than LB$\longrightarrow pvec2 = pvec1 > LB$
$M \longrightarrow$ array containing in each row a vector and in each column a parameter included in the objective function interval

Algorithm 6 Decreasing the ensemble size (Step 5.2)

```
for i = 1, 2, ..., n_θ  do
    Define the percentiles to compute ⟶ pct = [0 : 5 : 100]
    Compute the percentiles (pct) of each parameter along all the vectors ⟶ bins = percentile(M(:, i), pct)
    Save only those in even position⟶ total_bins = [total_bins; bins([2 : 2 : end])]
end for
for i = 1, 2, ..., n_θ do
    for j = 1, 2, ..., 10 do
        Find those with an absolute distance less than 1e − 4 ⟶ vector = find(|M(:, i) − total_bins(i, j)| < 1e − 4)
        Save ⟶ total_vector = [total_vector, vector]
    end for
end for
Select total_vector from M ⟶ Mr = M(total_vector)
Filter to be unique and sorted by its position
θ* ⟶ Best fit
cut-off⟶ 1e − 6
n_Mr ⟶ number of vectors in Mn
for i = 1, 2, ..., n_Mr do
    See if the parameter vector is different from all previous ones
    Flag variable to detect a different parameter ⟶ different_flag=1
    Temporary variable to loop over the already included vectors ⟶ index = 1
    while different_flag ==1 and index¡i do
        Compute the difference ⟶ d = sqrt(∑(M(index,:)/θ* − M(i,:)/θ*)^2)
        if (index != i) and d < 1e − 6 then
            Exit in case it is different ⟶different_flag=0
        end if
        index=index+1
    end while
    if different_flag==1 then
        Save the vector in the ensemble ⟶ Me=[Me, M(i,:)]
    end if
end for
Me ⟶ array of the vectors that are different between them
n_Me ⟶ number of vector in Me
Extract the selected vectors from the total ⟶ Ens = Me(randperm(n_Me, 1000))
```

Algorithm 7 Ensemble prediction computation and uncertainty quantification (Step 6)

```
Compute the prediction for each parameter vector contained in the ensemble ⟶ M_ens = simulate model for Ens
Compute the median for median for M_ens ⟶ M_ens_y = median(M_ens)
Compute the RMSE ⟶ RMSE = sqrt(∑((M_ens_y − ỹ)^2)/n_y)
Compute the NRMSE ⟶ NRMSE = RMSE/(max(ỹ) − min(ỹ))
```

2 Computational reproducibility and detailed results

We provide detailed results and the code to reproduce them at `https://doi.org/10.5281/zenodo.6782638`. For each case study, we provide the following file types:

- *.mlx*: notebooks in Matlab livescript format, implementing the pipeline described in the paper.
- *.html*: detailed reports for each case study, as produced by the Matlab livescripts.
- *.m*: MATLAB scripts with the main functions for the different steps of the pipeline
- *.mat*: additional files with the results of optimizations, experimental data, etc.
